# Supplementary material for: Mixed success for carbon payments and subsidies in support of forest restoration in the neotropics
Source: Nat Commun. 2023 Dec 15;14:8359. doi: 10.1038/s41467-023-43861-4 (PMC10724297; doi:10.1038/s41467-023-43861-4)
Supplement: Supplementary file 1 — Supplementary Information [file 41467_2023_43861_MOESM1_ESM.pdf]

Supplementary Materials

Mixed success for carbon payments and subsidies in support of forest restoration in the neotropics

Katherine Sinacore[1*], Edwin H. García[1,2], Alex Finkral[3], Michiel van Breugel[1,4,5], Omar R. Lopez[1,6], Carlos Espinosa[7], Andrea Miller[8], Theodore Howard[9], Jefferson S. Hall[10]

[1] Rohr Fellow, Agua Salud Project, Smithsonian Tropical Research Institute, Balboa, Ancón, Panamá, Panamá
[2] Facultad de Ciencias Naturales, Exactas y Tecnología, Universidad de Panamá, Ciencias Biológicas, Panamá, Panamá
[3] Eastwood Forests, Chapel Hill, North Carolina, USA
[4] Department of Geography, National University of Singapore, AS2-03-01, 1 Arts Link Singapore 117568
[5]Yale-NUS College, College Avenue West, Singapore, Singapore
[6] Instituto de Investigaciones Científicas y Servicios de Alta Tecnología (INDICASAT), Edificio 209, Clayton, Panamá
[7] Universidad Tecnológica de Panamá, Ancón, Panamá
[8] Sostante, Nature-Based Learning, Clayton, Panamá, Panamá
[9] University of New Hampshire, Durham, New Hampshire, USA
[10] ForestGEO, Agua Salud Project, Smithsonian Tropical Research Institute, Balboa, Ancón, Panamá, Panamá

*Corresponding author email: ksinacore@gmail.com
ORCID IDs:

KS: 0000-0002-8719-9248
MvB: 0000-0003-2778-7803
ORL: 0000-0003-4953-2123
JSH: 0000-0003-4761-9268

Supplementary Note 1: Timber species identified in the secondary forest network

Timber analyses here expand upon those presented in Hall et al[1]. Via annual inventories of the secondary forest plot network in Agua Salud, we identified 23 timber species, many of which were censused multiple times and were classified in different age groups. Not all species had enough individuals or a range of diameters to model projected growth to age 30. **Table S1** lists the species, the common name, the number of individuals, the maximum diameter at breast height (DBH, cm) and whether the species had sufficient individuals to be included in the model (next page).

Table S1. Timber species identified in secondary forest network plots. Genus and species name (Species), common name (Common name), number of individuals across secondary forest plots (No. ind.), maximum diameter at breast height (Max. DBH in centimeters) of each species and whether or not number of individuals was sufficient to meet modeling limitations.

Species	Common name	No. ind.	Max. DBH	Met modeling limitations?
Anacardium excelsum	Espavé, javillo, cornesuelo	8	53.1	Yes
Andira inermis	Almendro de río, harino, quira	5	39.6	No
Cedrela odorata	Cedro, cedro amargo, cedro cebolla	1	21.3	No
Chrysophyllum argenteum	Caimito de mono	11	26.2	Yes
Cordia allidora	Laurel, laurel negro, muñeco	24	30.1	Yes
Dedropanax arboreus	Vaquero	7	17	No
Enterolobium cyclocarpum	Corotú	1	71.3	No
Eschweilera amplexifolia		1	15.6	No
Eschweilera calyculata		1	13.5	No
Ficus insipida	Fig, higuerón	13	61.2	Yes
Genipa americana	Jagua	2	20.7	No
Gmelina arborea		3	19.8	No
Hieronyma alchorneoides	Zapatero, pilón, palo chancho, piedro, pantano	15	38	Yes
Ochroma pyramidale	Balso, balsa, lano	68	30.8	Yes
Ormosia macrocalyx	Alcornoque, coralillo, peronil, cabresto	1	10.5	No
Ormosio coccinea	Alcornoque, frijolito de la suerte, cabresto, coralillo, peronil	14	58.4	Yes
Schizolobium parahyba	Gallinazo, indio, tinecú, cigarillo, cucharo	2	60.7	No
Tabebuia guayacan	Guayacan	9	36.4	No
Tabebuia rosea	Roble, roble de sabana	2	37.1	No
Terminalia amazonia	Amarillo, roble amarillo, amarillo carabazuelo	188	58.4	Yes
Tetragastris panamensis	Animé, cuatro estomagos, chutra, kerosín	1	13.2	No
Vochysia ferruginea	Flor de mayo, botarrama, tecla, mayo	57	59.5	Yes
Zanthoxylum procerum	Arcabú, tachuelo, largarto	10	23.3	Yes

Supplementary Note 2: Tree and stand volume by species and land use

Based on data and analyses from García et al.[2] and Sinacore et al.[3], we measured tree aboveground biomass of 80 harvested trees of *Terminalia amazonia*, *Dalbergia retusa*, and *Pachira quinata*. Using the harvested data, the Smalian formula (García et al[2]) and a top diameter cut-off of 10 centimeters, we created species-specific tree volume equations for *T. amazonia* and *D. retusa* (**Table S2**). As no species nor site-specific volume equations existed for the other five enrichment species, we used our site-specific multi-species equation to calculate aboveground biomass (García et al[2]) and multiplied the aboveground biomass value by 0.5 to estimate tree volume (based on methods from Montagini et al[4]).

Table S2. Tree volume equations for all species. Formula shows the equation for calculating tree volume (m^3). Species, the formula of the equation, the variables (a-d) and the wood specific gravity are included for each species.

	Volume calculations					
**Species**	**Formula**	**a**	**b**	**c**	**d**	**WSG**
Anacardium excelsum	V = a + b(BD^2) + c(BD^3)	-0.003	0.000831	-0.00001902		
Byrsonima crassifolia	V = [[exp(a + (b*ln(BD^2)) + (c*ln(H)) + (d*(ln(WSG)))]*0.50/100	-4.04	0.88	1.48	0.75	0.53
Carapa guianensis	V = [[exp(a + (b*ln(BD^2)) + (c*ln(H)) + (d*(ln(WSG)))]*0.50/100	-4.04	0.88	1.48	0.75	0.43
Chrysophyllum argenteum	V = a + b(BD^2) + c(BD^3)	-0.003	0.000831	-0.00001902		
Cordia allidora	V = a + b(BD^2) + c(BD^3)	-0.003	0.000831	-0.00001902		
Dalbergia retusa	V = a + b(BD^2) + c(BD^3)	0.006779	0.000009488	0.000007838		
Dipteryx oleifera	V = [[exp(a + (b*ln(BD^2)) + (c*ln(H)) + (d*(ln(WSG)))]*0.50/100	-4.04	0.88	1.48	0.75	0.87
Ficus insipida	V = a + b(BD^2) + c(BD^3)	-0.003	0.000831	-0.00001902		
Hieronyma alchorneoides	V = [[exp(a + (b*ln(BD^2)) + (c*ln(H)) + (d*(ln(WSG)))]*0.50/100	-4.04	0.88	1.48	0.75	0.59
Ochroma pyramidale	V = a + b(BD^2) + c(BD^3)	-0.003	0.000831	-0.00001902		
Ormosio coccinea	V = a + b(BD^2) + c(BD^3)	-0.003	0.000831	-0.00001902		
Pachira quinata	V = a + b(BD^2)	0.005381	0.0003283			
Platymiscium pinnatum	V = [[exp(a + (b*ln(BD^2)) + (c*ln(H)) + (d*(ln(WSG)))]*0.50/100	-4.04	0.88	1.48	0.75	0.76
Tabebuia rosea	V = a + b(BD^2) + c(BD^3)	-0.003	0.000831	-0.00001902		
Terminalia amazonia	V = a + b(BD^2) + c(BD^3)	-0.003	0.000831	-0.00001902		
Vochysia ferruginea	V = a + b(BD^2) + c(BD^3)	-0.003	0.000831	-0.00001902		
Zanthoxylum procerum	V = a + b(BD^2) + c(BD^3)	-0.003	0.000831	-0.00001902		

Volume estimates were projected out to 30 years for all species where sufficient data were available using a Bayesian framework and nonlinear modeling. Figs S1-S3 show the actual data points in black, and the projected volumes based on the data. The enrichment planting and native species plantations were modeled by stand volume while the secondary forest data was first modeled at the individual tree-level and then multiplied by the number of individuals and calculated stand-volumes per hectare. For the native species plantation (Fig. S3), we projected volumes for all two-species mixtures and the five species mixture in addition to the five monoculture species. In the main text of the manuscript, we calculate the NPV of the monocultures. Some of the NPV's of the two-species mixtures are available in Sinacore et al[3]. Additionally, Mayoral et al[5] did not find that mixtures overyielded, except for the two-species mixture of *D. retusa* and *P. quinata*. The results of that combination can be found in Sinacore et al.[3]

Supplementary Note 3: Carbon dioxide equivalent estimates

Calculations for aboveground biomass (AGB) are explained in the main body of the manuscript and relied on locally derived equations for plantation trees scaled to stands[2], and trees for secondary forests[6] scaled to per ha basis[7]. AGB estimates for each land use were converted into carbon dioxide equivalent (CO_2e) in Mg per hectare. We projected these estimates out to year 30 (Table S3). Additionally, we used the annual growth in CO_2e Mg ha^{-1} and the market price for CO_2e ($18 per Mg) to estimate a variable carbon payment scenario (Fig S6).

Table S3. Carbon dioxide equivalent (CO_2e; megagrams per hectare) for each land use and treatment. Estimate is the mean CO_2e and the lower and upper values are the credible intervals around the estimate. Treatment codes are as follows: Ta – *Terminalia amazonia*, Dr – *Dalbergia retusa*, Ae – *Anacardium excelsum*, Pq – *Pachira quinata*, Tr – *Tabebuia rosea*, 5 spp mixtures (all five previous species together); Bc - *Byrosonima crassifolia,* Cg - *Carapa guianensis,* Dp – *Dipteryx oleifera,* Ha – *Hyeronima alchorneides,* Pp – *Platymiscium pinnatum*.

Land use	Treatment	Estimate	Lower	Upper
Secondary forest	Natural Regeneration	185.81	91.78	323.60
Native species planting	Ta mono	221.79	189.35	254.01
	Dr mono	33.72	28.42	39.12
	Ae mono	33.47	23.83	43.24
	Pq mono	33.61	26.69	40.91
	Tr mono	20.37	14.03	27.10
	5 spp mixtures	132.09	108.78	155.49
	Ae & Dr	34.14	24.56	44.76
	Ae & Pq	37.90	24.72	52.23
	Ae & Ta	181.09	149.68	210.94
	Ae & Tr	24.12	17.54	31.10
	Dr & Pq	50.19	40.49	60.62
	Dr & Ta	182.41	154.31	210.05
	Dr & Tr	33.82	25.64	41.85
	Pq & Ta	188.51	159.50	218.27
	Pq & Tr	21.09	15.83	26.57
	Ta & Tr	138.38	115.78	159.37
Enrichment planting	Bc enrichment	53.01	37.15	69.23
	Cg enrichment	48.98	33.98	64.60
	Dr enrichment	55.74	38.79	73.03
	Dp enrichment	55.39	38.19	73.41
	Ha enrichment	59.56	39.69	80.78
	Pp enrichment	48.20	34.16	62.65
	Ta enrichment	52.97	35.92	70.79

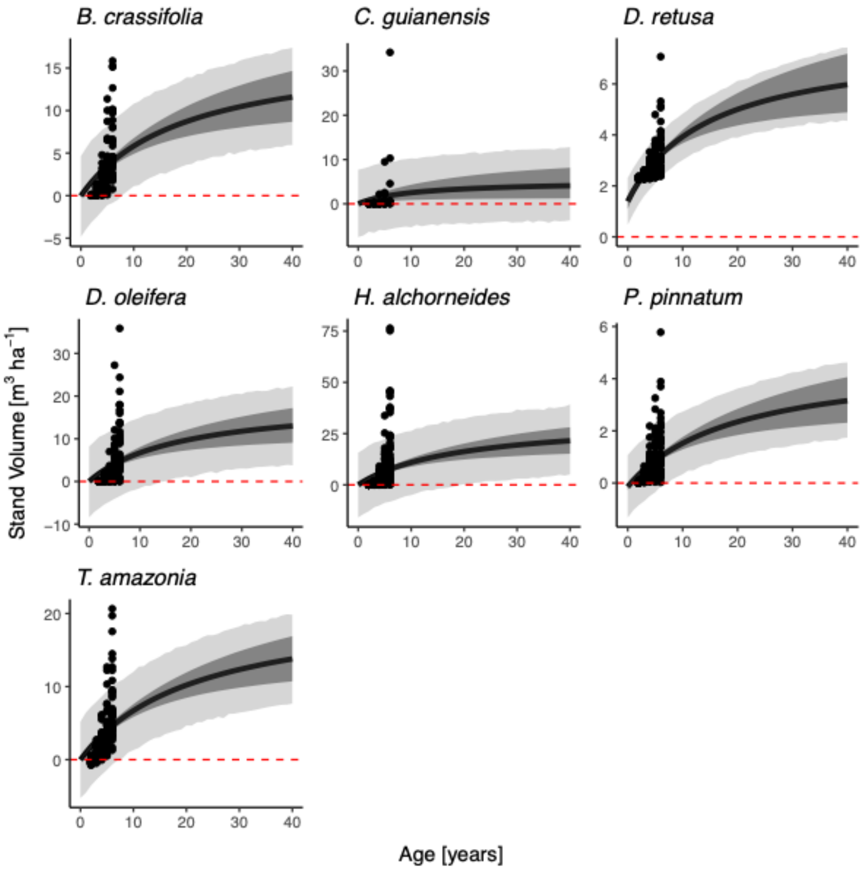

Fig. S1 Modeled stand-level volume (m^3 ha^{-1}) estimates by species over time for enrichment planting. Points represent real data points by plot. Solid black line represents predicted mean stand volume over time. Dark gray shading represents 95% credible interval and lighter gray shading represents 90% credible interval. Scientific names for each plot: *B. crassifolia – Byrosonima crassifolia, C. guianensis - Carapa guianensis, D. retusa – Dalbergia retusa, D. oleifera – Dipteryx oleifera, H. Alchorneides – Hyeronima alchorneides, P. pinnatum – Platymiscium pinnatum, T. amazonia – Terminalia amazonia*.

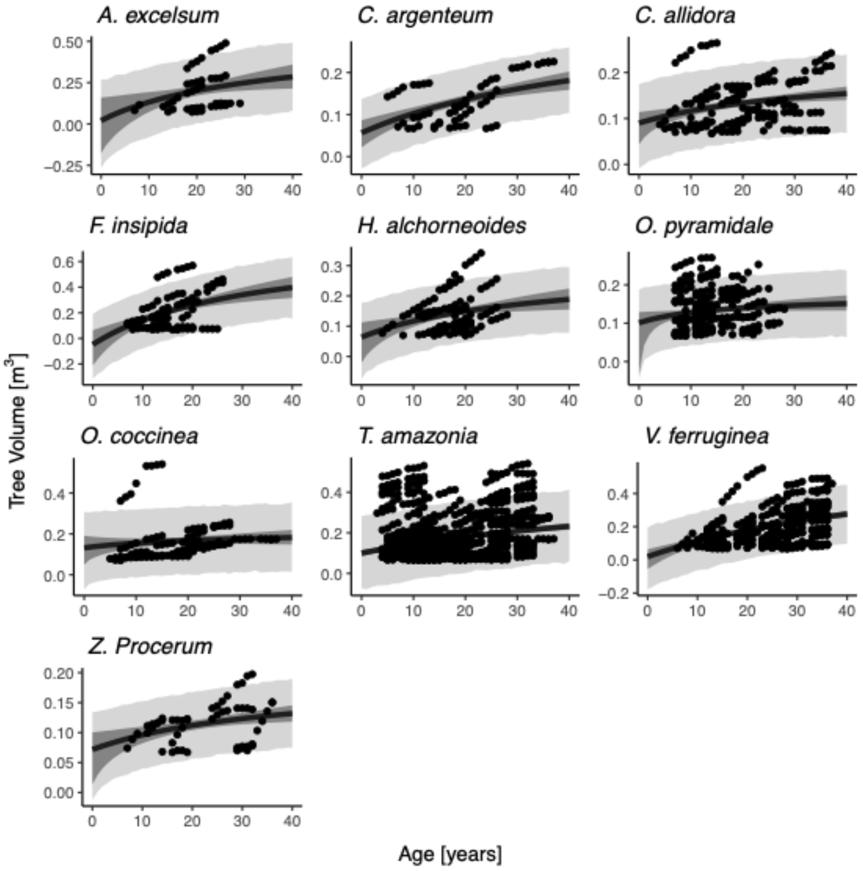

Fig. S2 Tree volume (m^3) modeled by age (years) for timber species within the secondary forest network for species with sufficient data to model tree volume over time. Points represent real data points by tree. Solid black line represents predicted mean tree volume over time. Dark gray shading represents 95% credible interval and lighter gray shading represents 90% credible interval. Scientific names at top of each plot: *A. excelsum – Anacardium excelusum, C. argenteum – Chrysolphyllum argenteum, C. allidora – Cordia allidora, F. insipida – Ficus insipida, H. alchorneoides – Hieronyma alchorneoides, O. pyramidale – Ochroma pyramidale, O. coccinea – Ormosio coccinea, T. amazonia – Terminalia amazonia, V. ferruginea – Vochysia ferruginea, Z. procerum – Zanthoxylum procerum*.

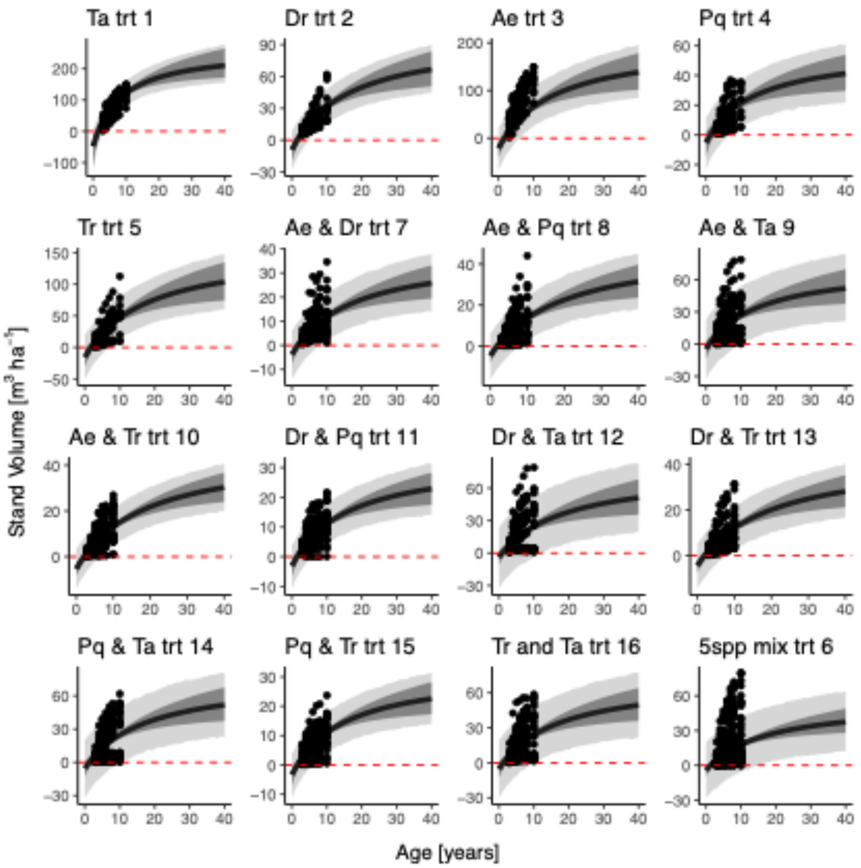

Fig. S3 Stand volume (m^3 ha^{-1}) modeled by age (years) for timber species within the native species plantations. Points represent real data points by site. Solid black line represents predicted mean tree volume over time. Dark gray shading represents 95% credible interval and lighter gray shading represents 90% credible interval. Trt refers to treatment and the initials of each species are in the title of each plot: Ta – *Terminalia amazonia*, Dr – *Dalbergia retusa*, Ae – *Anacardium excelsum*, Pq – *Pachira quinata*, and Tr – *Tabebuia rosea*. Treatments 7-16 are every combination of the five monoculture species (treatments 1-5). Treatment 6 is all five of those species together.

Net present value full datasets

Enrichment planting full data

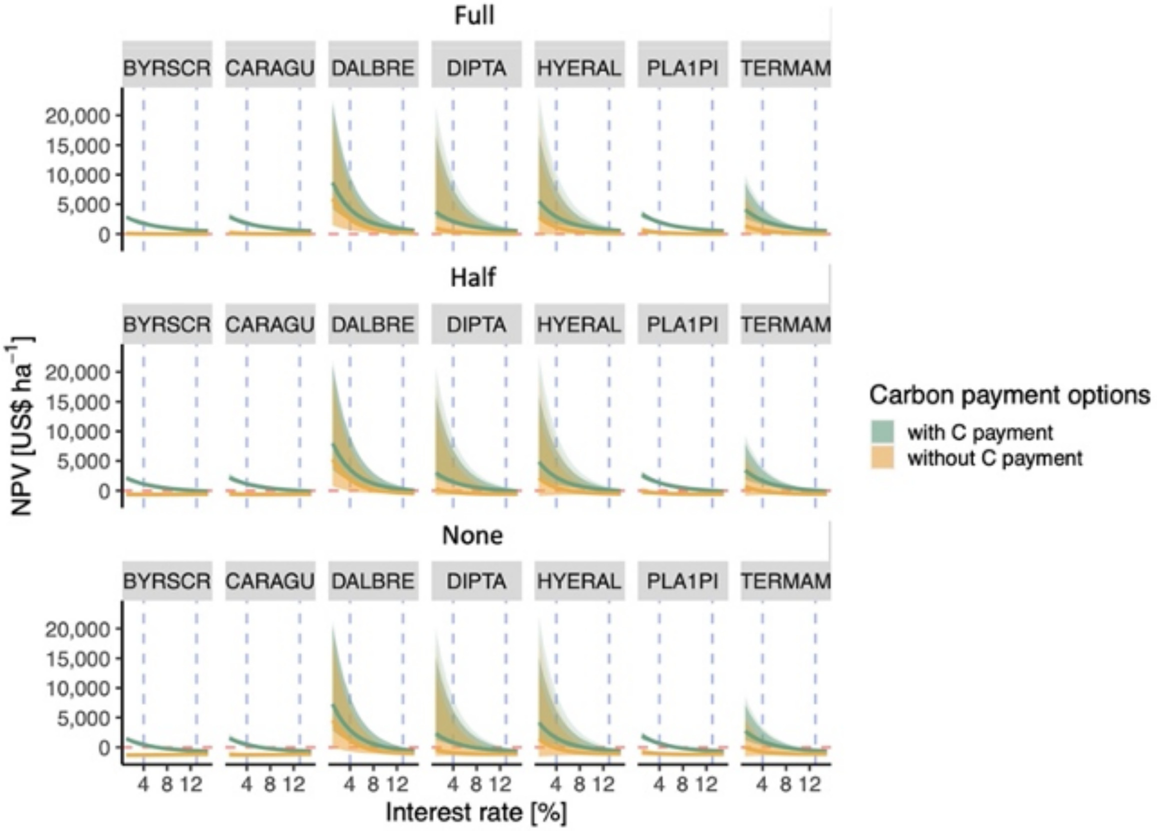

Fig. S4 Net present value (US$ ha^{-1}) by interest rate (%) for all enrichment species. NPV organized by financial support structure (full, half, none) and by carbon payment options (green: with payment; yellow: without payment. Scientific names for each plot: BYRSCR – *Byrosonima crassifolia,* CARAGU - *Carapa guianensis,* DALBRE – *Dalbergia retusa,* DIPTA – *Dipteryx oleifera,* HYERAL – *Hyeronima alchorneides,* PLA1PI – *Platymiscium pinnatum,* TERMAM – *Terminalia amazonia*. The red dashed lines represent where NPV is equal to zero. The vertical blue dashed lines represent an interest rate of 4% and 13%. Lighter shading represents 90% credible intervals and darker shading represents 95% credible intervals.

Native species plantation full data

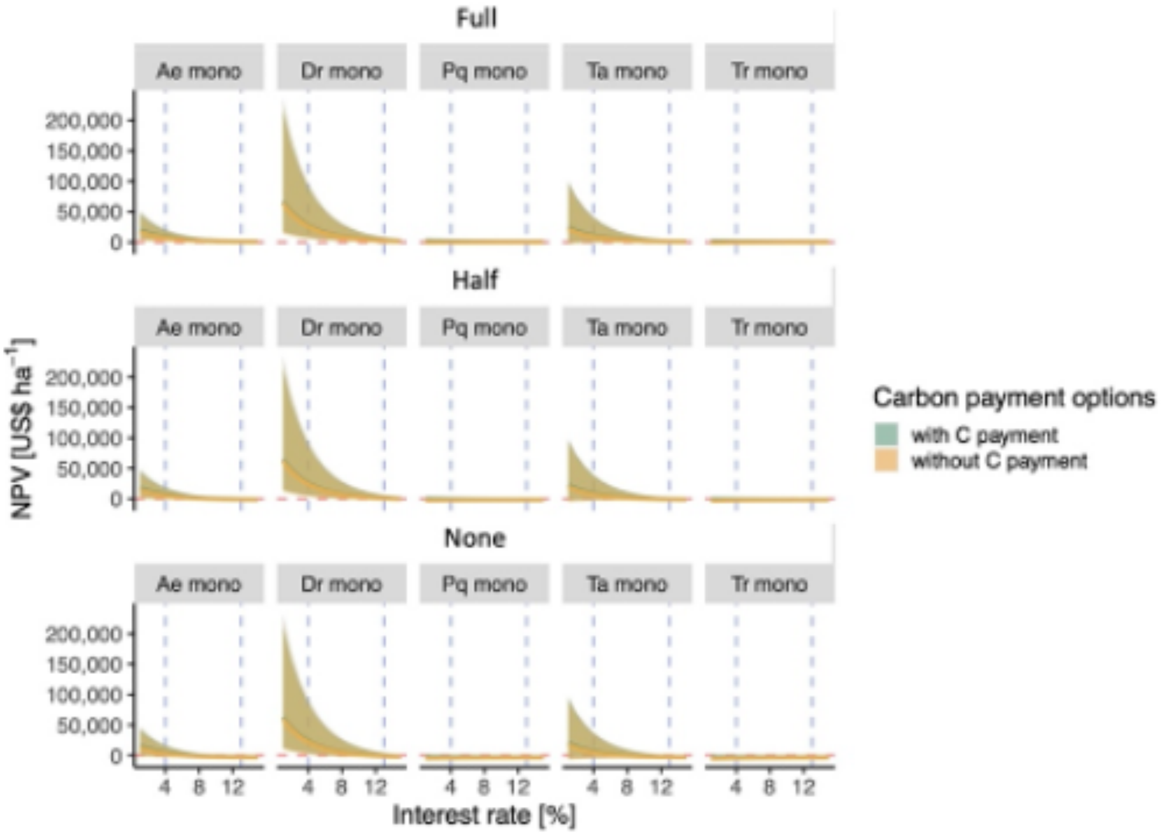

Fig. S5 Net present value (US$ ha^{-1}) by interest rate (%) for all native species plantings monocultures (mono). NPV organized by financial support structure (full, half, none) and by carbon payment options (green: with payment; yellow: without payment. Scientific names for each plot: Ae mono – *Anacardium excelsum*, Dr mono – *Dalbergia retusa*, Pq mono – *Pachira quinata*, Ta mono – *Terminalia amazonia* and Tr mono – *Tabebuia rosea*. The red dashed lines represent where NPV is equal to zero. The vertical blue dashed lines represent an interest rate of 4% and 13%. Lighter shading represents 90% credible intervals and darker shading represents 95% credible intervals.

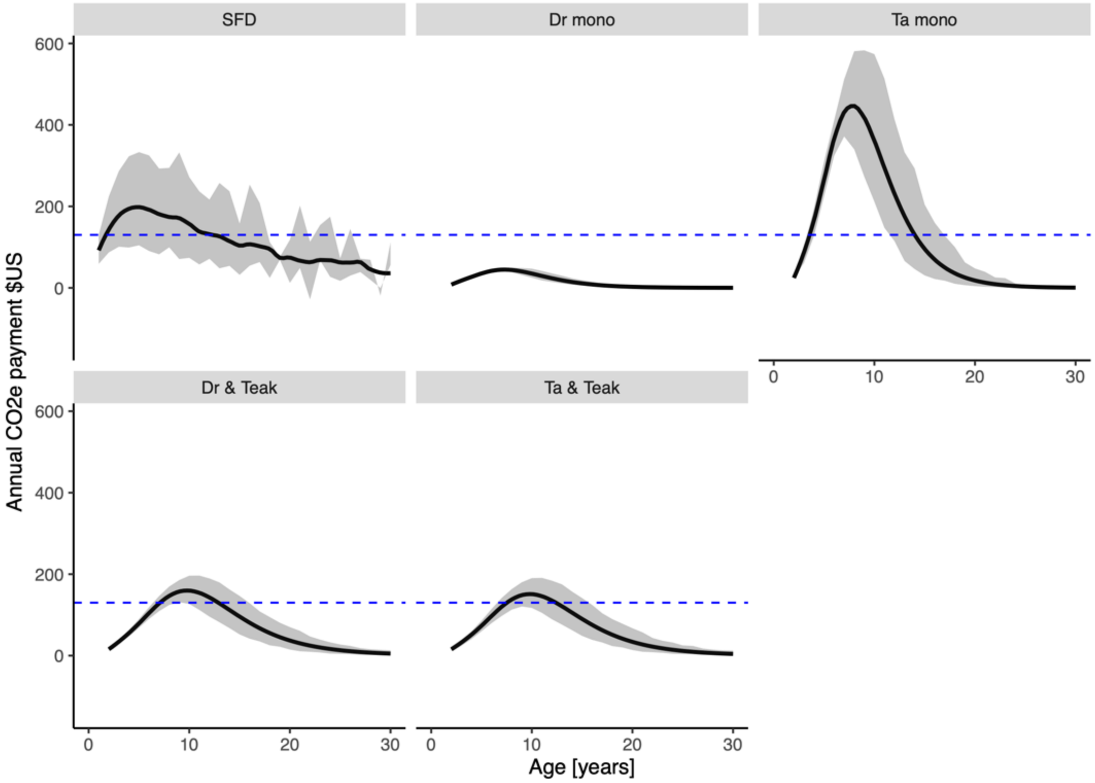

Fig. S6 Annual carbon dioxide equivalent (CO_2e) payments ($US) for focal land uses. Change in CO_2e payments based on the growth of CO_2e and the price per CO_2e over thirty years. Solid black lines represent the mean estimate of the payment, and the gray shading represents the 95% credible interval around the estimate. The dashed blue line represents the $130 flat annual payment. SFD – secondary forest, Dr mono – *Dalbergia retusa* in monocultures, Ta mono – *Terminalia amazonia* in monocultures, Dr & Teak – *D. retusa* and teak enrichment plantings, Ta & teak – *T. amazonia* and teak enrichment plantings.

Supplementary Note 4: Cattle estimates

We calculated the net present value of pasture and silvopasture from data collected by one of the authors (AM) as well as through personal conversations with landowners around the area of Agua Salud. To err on the side of a favorable estimate for cattle, we used the maximum capacity for cattle in the watershed in which we work of 1 cow per hectare in our calculations. Unlike some watersheds on fertile soils that can maintain 5 head per ha or more with silvopasture (e.g., [8,9]), we have not seen increased benefits beyond 1 head per ha. Generally, a cow can give birth to a calf every two years. Most of the male calves are sold at age 2, equivalent to US$ 300 per calf. Occasionally, when a calf is particularly robust, the owners will keep them and raise them for 5 years. The owners estimate they raise on of these more robust calves every 5 years and they are sold for $600. The cost of raising the cows includes vaccines, feed, salt, and building and maintaining fencing. Based on the project's pasture and silvopasture costs, we estimate that the cost per year to maintain silvopasture is $200 and the cost per year to maintain pasture is $100. We assume no establishment costs in this calculation as we work on existing pastures. Thus, were land to be cleared and pasture planted, costs would go up considerably. We estimated the NPV of silvopasture and pasture based on this information and using Equation 1 from the main manuscript (Table 4). Our costs and revenues fall within the range found in Stefanski et al[10] (conducted in similar area) and Grado et al[11]. Note that the Panama Canal Authority subsidizes silvopasture by paying for system establishment and maintenance for the first three years[12].

Table S4. Sensitivity analysis of traditional cattle and silvopasture. Net present values (NPVs, $US) are estimated per hectare with varying cattle density and interest rates (%) by cattle system – Pasture (tranditional pasture) and silvopasture.

	1 cow per hectare	0.87 cows per ha	0.435 cows per ha	1 cow per hactare	0.85 cows per hectare	2 cows per ha
	Pasture			Silvopasture		
Interest rate	NPV ($US per ha)					
1	2803.97	2439.45	1219.73	223.20	189.72	446.40
2	2407.05	2094.13	1047.07	167.41	142.29	334.81
3	2086.45	1815.21	907.60	126.40	107.44	252.81
4	1825.05	1587.79	793.90	95.85	81.47	191.69
5	1609.93	1400.64	700.32	72.69	61.79	145.38
6	1431.28	1245.22	622.61	54.80	46.58	109.60
7	1281.59	1114.98	557.49	40.68	34.58	81.37
8	1155.06	1004.90	502.45	29.28	24.89	58.57
9	1047.23	911.09	455.54	19.86	16.88	39.72
10	954.58	830.49	415.24	11.89	10.11	23.78
11	874.39	760.72	380.36	5.01	4.26	10.02
12	804.47	699.89	349.94	-1.05	-0.89	-2.10
13	743.09	646.49	323.24	-6.48	-5.50	-12.95
14	688.87	599.32	299.66	-11.40	-9.69	-22.79
15	640.69	557.40	278.70	-15.91	-13.52	-31.82

References

1. Hall, J. S., Sinacore, K., García, E. H. & Van Breugel, M. Seeking forestry-based win-win solutions to combat climate change and improve rural livelihoods in central Panama. *Proceedings of the World Forestry Conference* (2020).
2. García, E. H., Sinacore, K., Hall, J. S. & Lopez, O. R. Allometric equations for three tropical timber species in central Panama. (Universdad de Panama, 2022).
3. Sinacore, K. *et al.* Towards effective reforestation: growth and commercial value of four commonly planted tropical timber species on infertile soils in Panama. *New Forests* (2022) doi:10.1007/s11056-022-09906-0.
4. Petit, B. & Montagnini, F. Growth equations and rotation ages of ten native tree species in mixed and pure plantations in the humid neotropics. *Forest Ecology and Management* **199**, 243–257 (2004).
5. Mayoral, C., van Breugel, M., Cerezo, A. & Hall, J. S. Survival and growth of five Neotropical timber species in monocultures and mixtures. *Forest Ecology and Management* **403**, 1–11 (2017).
6. van Breugel, M., Ransijn, J., Craven, D., Bongers, F. & Hall, J. S. Estimating carbon stock in secondary forests: Decisions and uncertainties associated with allometric biomass models. *Forest Ecology and Management* **262**, 1648–1657 (2011).
7. Hall, J. S. *et al.* Deforestation scenarios show the importance of secondary forest for meeting Panama's carbon goals. *Landscape Ecology* **37**, 673–694 (2022).
8. Murgueitio, E., Calle, Z., Uribe, F., Calle, A. & Solorio, B. Native trees and shrubs for the productive rehabilitation of tropical cattle ranching lands. *Forest Ecology and Management* **261**, 1654–1663 (2011).
9. Chizmar, S. *et al.* A discounted cash flow and capital budgeting analysis of silvopastoral systems in the Amazonas region of Peru. *Land* **9**, 1–15 (2020).
10. Stefanski, S. F., Shi, X., Hall, J. S., Hernandez, A. & Fenichel, E. P. Teak–cattle production tradeoffs for Panama Canal Watershed small scale producers. *Forest Policy and Economics* **56**, 48–56 (2015).
11. Grado, S. C., Hovermale, C. H. & St. Louis, D. G. A financial analysis of a silvopasture system in southern Mississippi. *Agroforestry Systems* **53**, 313–322 (2001).
12. Hall, J. S., Kirn, V., Yanguas-Fernandez, & (Eds.). Managing Watersheds for Ecosystem Services in the Steepland Neotropics. in *Inter-American Development Bank Monograph* 186 (2015).
