# Supplementary material for: Mixed success for carbon payments and subsidies in support of forest restoration in the neotropics
Source: Nat Commun. 2023 Dec 15;14:8359. doi: 10.1038/s41467-023-43861-4 (PMC10724297; doi:10.1038/s41467-023-43861-4)
Supplement: Supplementary file 2 — Reporting Summary [file 41467_2023_43861_MOESM2_ESM.pdf]

nature portfolio

Corresponding author(s): Katherine Sinacore

Last updated by author(s): Nov 2, 2023

Reporting Summary

Nature Portfolio wishes to improve the reproducibility of the work that we publish. This form provides structure for consistency and transparency in reporting. For further information on Nature Portfolio policies, see our Editorial Policies and the Editorial Policy Checklist.

Statistics

For all statistical analyses, confirm that the following items are present in the figure legend, table legend, main text, or Methods section.

n/a	Confirmed	
☐	☒	The exact sample size (n) for each experimental group/condition, given as a discrete number and unit of measurement
☐	☒	A statement on whether measurements were taken from distinct samples or whether the same sample was measured repeatedly
☒	☐	The statistical test(s) used AND whether they are one- or two-sided<br>*Only common tests should be described solely by name; describe more complex techniques in the Methods section.*
☒	☐	A description of all covariates tested
☐	☒	A description of any assumptions or corrections, such as tests of normality and adjustment for multiple comparisons
☐	☒	A full description of the statistical parameters including central tendency (e.g. means) or other basic estimates (e.g. regression coefficient) AND variation (e.g. standard deviation) or associated estimates of uncertainty (e.g. confidence intervals)
☒	☐	For null hypothesis testing, the test statistic (e.g. F, t, r) with confidence intervals, effect sizes, degrees of freedom and P value noted<br>*Give P values as exact values whenever suitable.*
☐	☒	For Bayesian analysis, information on the choice of priors and Markov chain Monte Carlo settings
☒	☐	For hierarchical and complex designs, identification of the appropriate level for tests and full reporting of outcomes
☒	☐	Estimates of effect sizes (e.g. Cohen's d, Pearson's r), indicating how they were calculated

Our web collection on statistics for biologists contains articles on many of the points above.

Software and code

Policy information about availability of computer code

Data collection	Data collected by traditional forest inventory methods (diameter tapes, height poles). No software used.
Data analysis	R version 4.2.0 was used for all data analysis

For manuscripts utilizing custom algorithms or software that are central to the research but not yet described in published literature, software must be made available to editors and reviewers. We strongly encourage code deposition in a community repository (e.g. GitHub). See the Nature Portfolio guidelines for submitting code & software for further information.

Data

Policy information about availability of data

All manuscripts must include a data availability statement. This statement should provide the following information, where applicable:

- Accession codes, unique identifiers, or web links for publicly available datasets
- A description of any restrictions on data availability
- For clinical datasets or third party data, please ensure that the statement adheres to our policy

Data and code are included in the manuscript submission with codes and data available on OSF: https://osf.io/3gqz2/?view_only=e992606bc46f4499ad49b3288f60455b

Research involving human participants, their data, or biological material

Policy information about studies with human participants or human data. See also policy information about sex, gender (identity/presentation), and sexual orientation and race, ethnicity and racism.

Reporting on sex and gender	NA
Reporting on race, ethnicity, or other socially relevant groupings	NA
Population characteristics	NA
Recruitment	NA
Ethics oversight	NA

Note that full information on the approval of the study protocol must also be provided in the manuscript.

Field-specific reporting

Please select the one below that is the best fit for your research. If you are not sure, read the appropriate sections before making your selection.

☐ Life sciences ☐ Behavioural & social sciences ☒ Ecological, evolutionary & environmental sciences

For a reference copy of the document with all sections, see nature.com/documents/nr-reporting-summary-flat.pdf

Ecological, evolutionary & environmental sciences study design

All studies must disclose on these points even when the disclosure is negative.

Study description	The secondary forest network consists of 108 plots established across a chronosequence in 2008. The native species plantations consist of two blocks, with 267 plots total established in 2008. The enrichment planting had a total of 245 plots established in 2018. The Agua Salud Project (where the study is based) is a 700 ha experiment. Within those boundaries, we were limited by space, available land, and forest age in determining the sample size. Even so, this is the largest replicated study of this kind in the tropics.
Research sample	We worked with data produced by the Agua Salud Project. It is the only project in the tropics that has repeated measurements of secondary forest (of different age classes) and tree plantations.
Sampling strategy	Plots were replicated across a 700 hectare study area with randomization of plot locations. For sites where trees are planted, the location of each species was also randomized. We used data from all the sites for our analyses.
Data collection	Tree diameters and heights were collected by a team of technicians, annually from 2008 to 2018 for the secondary forest and native species, and teak; and since 2016 for the enrichment planting (see detail below).
Timing and spatial scale	The inventories were done annually, during the rainy season. The exception was that the native species plantation and the secondary forest were not inventoried in 2016 and 2017 respectively, due to lack of funding those years.
Data exclusions	For the enrichment planting, two sites were excluded because the landowners the land either burned because of a fire or because the landowner let cattle back on to the land in the middle of the study.
Reproducibility	This is a long term research site with annual inventories (over 1.1 million individual tree measurements total). We excluded two enrichment planting sites because the land either bured for the landowner let cattle into the site during the study. We cross-checked our estimated data with other studies that calculated tree volumes.
Randomization	Plot distribution was random for all sites. Enrichment planting also had randomized blocks of native species planted within.
Blinding	Blinding was included in the study because the outcome measure (tree size and growth) was quantitative and objective.

Did the study involve field work? ☒ Yes ☐ No

Field work, collection and transport

Field conditions	Daily temperatures range from 23-32 C on average. Mean annual rainfall is 2700m. During the 2015-2016 El Nino, there was 50% less rainfall than normal
Location	The different research sites are located within the Agua Salud Project of the Smithsonian Tropical Research Institute in Panama (9 13’N, 79 47’W 330 m amsl).

Access & import/export	We have ongoing support and permits from MiAmbiente (similar to the USA's EPA), to work in Panama.
Disturbance	The Agua Salud project is located within the Panama Canal Watershed, and near local landowners. We ensured that we did not affect their land during the field work.

Reporting for specific materials, systems and methods

We require information from authors about some types of materials, experimental systems and methods used in many studies. Here, indicate whether each material, system or method listed is relevant to your study. If you are not sure if a list item applies to your research, read the appropriate section before selecting a response.

Materials & experimental systems

n/a	Involved in the study	
☒	☐	Antibodies
☒	☐	Eukaryotic cell lines
☒	☐	Palaeontology and archaeology
☒	☐	Animals and other organisms
☒	☐	Clinical data
☒	☐	Dual use research of concern
☐	☒	Plants

Methods

n/a	Involved in the study	
☒	☐	ChIP-seq
☒	☐	Flow cytometry
☒	☐	MRI-based neuroimaging

Dual use research of concern

Policy information about dual use research of concern

Hazards

Could the accidental, deliberate or reckless misuse of agents or technologies generated in the work, or the application of information presented in the manuscript, pose a threat to:

No	Yes	
☒	☐	Public health
☒	☐	National security
☒	☐	Crops and/or livestock
☒	☐	Ecosystems
☒	☐	Any other significant area

Experiments of concern

Does the work involve any of these experiments of concern:

No	Yes	
☒	☐	Demonstrate how to render a vaccine ineffective
☒	☐	Confer resistance to therapeutically useful antibiotics or antiviral agents
☒	☐	Enhance the virulence of a pathogen or render a nonpathogen virulent
☒	☐	Increase transmissibility of a pathogen
☒	☐	Alter the host range of a pathogen
☒	☐	Enable evasion of diagnostic/detection modalities
☒	☐	Enable the weaponization of a biological agent or toxin
☒	☐	Any other potentially harmful combination of experiments and agents

Plants

Seed stocks	For the tree planting (native species plantation, teak plantation, and enrichment) seedlings were provided by seeding nurserys in Panama. The secondary forest is natural rengeneration.
Novel plant genotypes	NA
Authentication	NA
